# Resveratrol Treatment Prevents Increase of Mast Cells in Both Murine OVA Enteritis and IL-10^−/−^ Colitis

**DOI:** 10.3390/ijms23031213

**Published:** 2022-01-21

**Authors:** Sabrina Bilotta, Julian Arbogast, Nadine Schart, Maurice Frei, Axel Lorentz

**Affiliations:** Institute of Nutritional Medicine, University of Hohenheim, Fruwirthstraße 12, 70599 Stuttgart, Germany; sabrina.bilotta@uni-hohenheim.de (S.B.); julian.arbogast@uni-hohenheim.de (J.A.); nadine.schart@uni-hohenheim.de (N.S.); maurice.frei@uni-hohenheim.de (M.F.)

**Keywords:** mast cells, food allergy, enteritis, IBD, colitis, resveratrol, polyphenols, nutraceuticals

## Abstract

Mast cells are involved in allergic and other inflammatory diseases. The polyphenol resveratrol is known for its anti-inflammatory properties and may be used as nutraceutical in mast cell associated diseases. We analyzed the effect of resveratrol on mast cells in vivo in ovalbumin-induced allergic enteritis as well as experimental colitis in IL-10^−/−^ mice which received resveratrol via drinking water. Treatment with resveratrol prevented the increase in mast cells in both allergic enteritis and chronic colitis in duodenum as well as in colon. Further, it delayed the onset of diseases symptoms and ameliorated diseases associated parameters such as tissue damage as well as inflammatory cell infiltration in affected colon sections. In addition to the findings in vivo, resveratrol inhibited IgE-dependent degranulation and expression of pro-inflammatory cytokines such as TNF-α in IgE/DNP-activated as well as in LPS-activated bone marrow-derived mast cells. These results indicate that resveratrol may be considered as an anti-allergic and anti-inflammatory plant-derived component for the prevention or treatment of mast cell-associated disorders of the gastrointestinal tract.

## 1. Introduction

The prevalence of gastrointestinal disorders such as food allergies or inflammatory bowel disease (IBD) has increased in western countries throughout the past decades [1,2]. Pharmaceutic medication can be associated with negative side effects such as vomiting or nausea for patients affected [3,4,5]. Although conventional drug therapy is well established and safe [6], generally low compliance has been reported [7,8,9,10,11,12,13]. Reasons for low compliance may be high costs, risk of adverse effects or long treatment durations [14]. Natural medication may be associated with none or less adverse effects [4]. Plant substances such as stilbenes, flavonoids and others may be a good alternative and additive therapy option to treat the clinical symptoms of allergy [15,16]. In the context of nutraceuticals, polyphenols such as resveratrol could be of special interest in the near future due to their wide beneficial immunomodulatory effects [17].

Mast cells (MC) are key effector cells of type I allergy and release pro-inflammatory mediators such as pre-stored histamine or de novo synthesized cytokines and lipid mediators in response to IgE-dependent stimulation via the FcεRI receptor crosslinking by antigens [18]. The role of MC and their mediators was numerously shown not only in vitro in different MC models [19,20,21] or in vivo in several allergic conditions [22,23,24] but also in humans [25,26,27]. Consequently, MC play an important role in gastrointestinal disorders due to food allergens [28]. Additionally, MC were shown to be involved in IBD [29,30,31,32] of which ulcerative colitis and Crohn’s disease represent the most common disorders [32]. For example, the MC proteases MCPT-6 and Prss31 were found to be involved in the formation of acute colitis [33,34,35] and MC numbers were found to be elevated in IBD patients and mice with colitis [36,37].

Resveratrol (trans-3,4′,5 trihydroxystilbene, trans-Resveratrol) is mainly found in grapes, berries or peanuts [38,39]. Numerous studies already showed the anti-inflammatory effects of resveratrol in the context of allergy and MC reactivity [24,40,41,42,43,44]. We have recently shown that resveratrol is a potent inhibitor of primary human intestinal mast cells’ (hiMC) activity isolated from surgical tissue from patients undergoing bowel resection [40]. The anti-inflammatory effect of resveratrol was not only shown in vitro [24,40] but also in vivo in murine models of airway inflammation, atopic dermatitis or allergic rhinitis [41,42,43,44]. This was confirmed in humans by intranasal administration of resveratrol in children and adults suffering from allergic rhinitis [25,26]. 

Resveratrol treatment was found to attenuate clinical symptoms of ovalbumin (OVA)-induced allergic rhinitis in mice accompanied by a reduction in the release of inflammatory mediators [45]. Further, resveratrol treatment resulted in reduced cytokine concentrations in bronchoalveolar fluid (BALF) in mice with OVA-induced asthma [46]. Moreover, anti-allergic property of resveratrol was shown in OVA-induced model of food allergy by preventing onset of allergic symptoms as well as reduced IgE serum production [47]. Food allergy affects the gastrointestinal tract including local inflammations. These inflammations are amongst others associated with infiltration of immune cells such as MC into affected tissues [28,48,49]. In addition, intestinal inflammation such as IBD was also recognized to be correlated with MC numbers and activity [30,31,50]. We observed an increased number of MC and higher expression of MC proteases in IL-10^−/−^ mice which develop a spontaneous form of chronic colitis due to the missing anti-inflammatory cytokine IL-10 [51,52,53]. Thus, we examined the immunomodulatory role of resveratrol on MC in both an OVA-induced allergic enteritis model and the IL-10^−/−^ mouse.

## 2. Results

### 2.1. Resveratrol Inhibits Increase in MC in OVA-Enteritis

**Figure 1:**
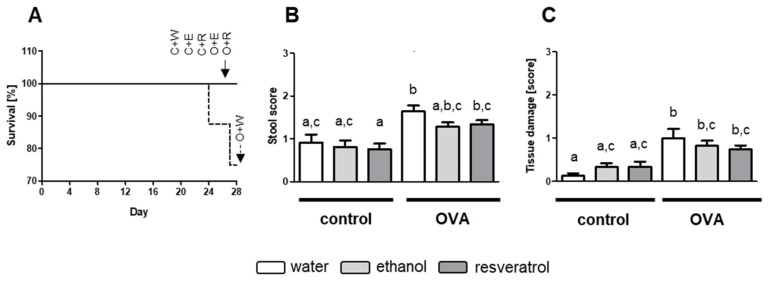
(**A**) Survival rate (%) of mice after 28 days, (**B**) stool score after first signs of diarrhea after 4th gavage of ovalbumin (OVA) and (**C**) scores for tissue damage in colon. Mice received water (white, (W)), 50 mg/kg bodyweight (BW) ethanol (light grey, (E)) or 50 mg/kg BW resveratrol (dark grey, (R)) via drinking water for 28 days and were intraperitoneally (i.p.) sensitized with 50 µg OVA in alum (1:2) on day 5 and 11 and further treated with or without (control, (**C**)) 50 mg OVA orally on day 15, 18, 20, 22, 24 and 27. Scores were determined in at least three hematoxylin and eosin (H&E) stained tissue sections, group size was n = 8, respectively. Values are mean ± SEM. Common letters indicate no significant difference between groups, different letters indicate significant change with at least *p* < 0.05. *p*-values for the respective data sets are shown in Figure S1.

To induce allergic enteritis, animals were intraperitoneally (i.p.) sensitized twice with 50 µg and challenged six times with 50 mg OVA orally. Allergic disorders in the gut due to Th2 immune responses are known to be accompanied with diarrhea [28]. None of the animals receiving OVA showed signs of severe diarrhea (score 3), but overall stool score was significantly elevated in comparison to controls. First signs of stool softening appeared after gavage number 4 on experimental day 22. Overall, stool scores for OVA groups were significantly elevated in comparison to control animals (Figure 1A). In OVA-challenged mice, stool scores were the highest for OVA group receiving no additive. Ethanol and resveratrol receiving OVA groups thereby showed less high stool scores than those without additive and these observations were not different from effects observed in control groups (Figure 1A). Two animals of the OVA group receiving water died of an anaphylactic shock, after fifth and sixth OVA application, respectively, whereas all animals treated with resveratrol or the vehicle ethanol survived (Figure 1B). 

Food allergy and enteritis are accompanied by histological changes and injury in the gut [50,54,55,56,57]. We found that OVA-induced allergic enteritis resembles a mild form of inflammation. All determined scores were relatively low (Figure 1C). Although resveratrol administration seemed to attenuate tissue damage, the results were not significant (Figure 1C). Nonetheless, in OVA-treated animals receiving the additive resveratrol, levels were diminished down to the levels of the control mice. 

**Figure 2:**
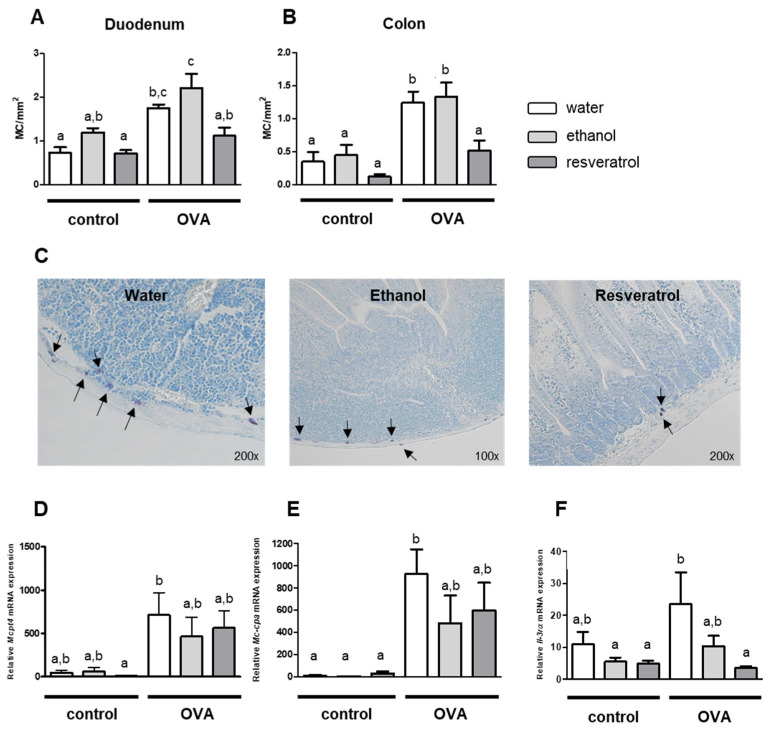
Mast cell (MC) numbers per mm^2^ in duodenum (**A**) and colon (**B**) tissue and representative pictures of MC in duodenum of OVA-treated mice (**C**). mRNA expression of *Mcpt4* (**D**) (n = 8), *Mc-cpa* (**E**) (n = 5) and *Il-3rα* (**F**) (n = 6–8) in colon. Mice received water (white), 50 mg/kg BW ethanol (light grey) or 50 mg/kg BW resveratrol (dark grey) via drinking water for 28 days and were i.p. sensitized with 50 µg OVA in alum (1:2) on day 5 and 11 and further treated with or without (control) 50 mg OVA orally on day 15, 18, 20, 22, 24 and 27. Numbers of MC were determined in toluidine blue stained duodenum and colon sections, respectively and are indicated by black arrows (**C**), group size was n = 8, respectively. Values are mean ± SEM. Common letters indicate no significant difference between groups, different letters indicate significant change with at least *p* < 0.05. *p*-values for the respective data sets are given in Figure S2.

The involvement of MC in enteritis induced by oral allergens was reported earlier [28]. Here, we found a significant increase in MC numbers in duodenum and colon after OVA challenge (Figure 2A,B). We observed that in OVA-induced allergic enteritis, MC numbers were strongly decreased in response to treatment with resveratrol. This observation was made in the colon (Figure 2B) as well as in the duodenum (Figure 2A). Thereby, a higher MC number was detected per mm^2^ in the duodenum of the mice receiving OVA in direct comparison to colon sections. Exemplary pictures of MC in duodenum tissue of OVA-treated mice are shown in Figure 2C. Noteworthy, resveratrol diminished MC in both GIT sections down to a level of the control mice not receiving OVA (Figure 2A,B). Expression of MC proteases such as MCPT4 and MC-CPA was also strongly increased in intestinal tissues of OVA-treated mice (Figure 2D,E). However, we did not detect significant inhibitory effects of resveratrol on mRNA expression of MC proteases in comparison to the control group receiving the vehicle ethanol. In addition to increased MC numbers and increased expression of MC proteases, we found an increased expression of the receptor for the murine MC growth factor IL-3 [58] in OVA-induced enteritis. Remarkably, the expression of the α-chain of the IL-3 receptor was strongly down-regulated in response to treatment with resveratrol (Figure 2F). This finding can explain the inhibitory effect of resveratrol on MC in OVA enteritis.

### 2.2. Resveratrol Inhibits Increase in MC and Shows Anti-Inflammatory Effects in IL-10^−/−^ Mice

**Figure 3:**
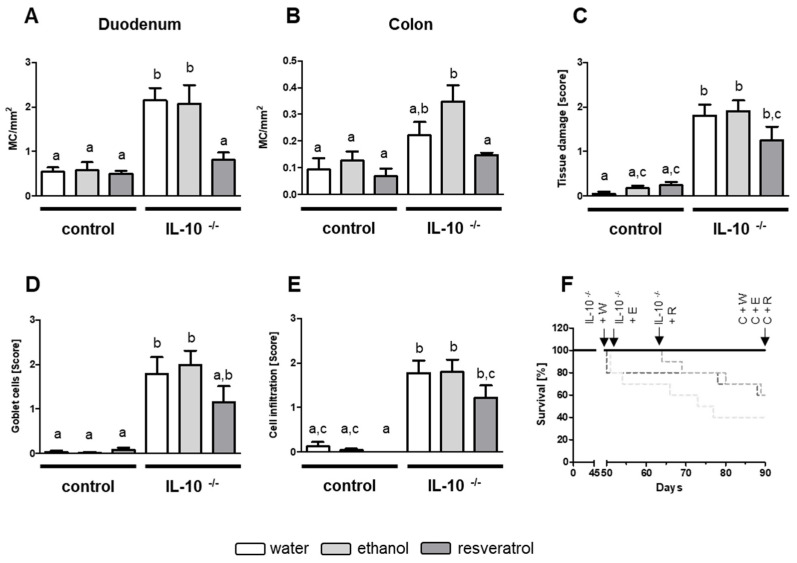
MC numbers per mm^2^ in duodenum (**A**) and colon (**B**) tissue samples and scores for (**C**) tissue damage, (**D**) goblet cell numbers and (**E**) cell infiltration in colon as well as (**F**) survival rate [%] of mice after 90 days. Mice received water (white, (W)), 50 mg/kg BW ethanol (light grey, (E)) or 50 mg/kg BW resveratrol (dark grey, (R)) via drinking water for 90 days. Scores were determined in at least three H&E-stained tissue sections. MC numbers were counted in toluidine blue stained duodenum and colon sections, respectively. Group size was n = 5 for BALB/c mice (controls, (C)) and n = 9–10 for IL-10^−/−^, respectively. Values are mean ± SEM. Common letters indicate no significant difference between groups, different letters indicate significant change with at least *p* < 0.05. *p*-values for the respective data sets are given in Figure S3.

As increase in MC numbers in OVA-induced allergic enteritis was prevented by resveratrol application, we further aimed to check for resveratrol effects in experimental colitis of IL-10^−/−^ mice. It was previously shown that MC are enhanced in patients’ inflamed tissue suffering from IBD [30,31,50]. Moreover, Hamilton et al. supposed that MC tryptase could play a critical pro-inflammatory role in IBD [33]. We could show that, like in OVA-induced enteritis, an increase in MC numbers takes place in the duodenum and colon of IL-10^−/−^ mice (Figure 3A,B). Again, treatment with resveratrol prevented the increase in MC numbers in duodenum and colon sections of IL-10^−/−^ mice (Figure 3A,B). Colitis-related parameters such as tissue damage (Figure 3C), reduction in goblet cell numbers (Figure 3D) as well as immune cell infiltration (Figure 3E) in colon were significantly elevated in IL-10^−/−^ mice and lowered by resveratrol treatment. In all cases, the scores of resveratrol receiving IL-10^−/−^ mice did not significantly differ from the levels of the control wildtype groups. Overall, 6 IL-10^−/−^ mice (40%) receiving ethanol as well as 4 animals (60%) receiving no additive developed signs of severe colitis and had to be removed from the study due to reaching a single end point score of 2 or a cumulative score of ≥3. IL-10^−/^^−^ group receiving resveratrol showed a similar survival rate of 60%, but it is noteworthy that onset of colitis and end point scores for these animals were achieved clearly later (day 64) in the experimental time course compared to control IL-10^−/−^ groups receiving no additive (day 50) or ethanol (day 51) (Figure 3F).

### 2.3. Resveratrol Inhibits Degranulation and Expression of Pro-Inflammatory Cytokines in BMMC

**Figure 4:**
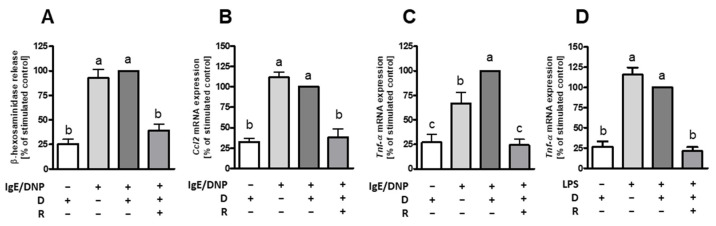
Degranulation, chemokine and cytokine mRNA expression in mouse bone marrow-derived mast cells (BMMC) following treatment with resveratrol. Cells were incubated with 50 µM resveratrol (R) or the corresponding vehicle control DMSO (D) for 60 min prior to 2,4-dinitrophenyl (DNP)-specific IgE treatment for 60 min and subsequent stimulation with 10 µg/mL DNP for 30 min to determine β-hexosaminidase release (**A**) (n = 14) and 90 min for mRNA expression of *Ccl2* (**B**) (n = 3) and *Tnf-α* (**C**) (n = 10). For lipopolysaccharide (LPS) stimulation, cells were incubated with 50 µM resveratrol or the corresponding control DMSO for 60 min prior to treatment with 1 µg/mL LPS for 3 h to determine *Tnf-α* mRNA expression (**D**) (n = 13). Values are mean ± SEM. Common letters indicate no significant difference between groups, different letters indicate significant change with at least *p* < 0.05. *p*-values for the respective data sets are given in Figure S4.

We recently reported that resveratrol inhibits activation of hiMC [40]. As the increase in MC numbers was prevented in duodenum and colon of mice in OVA enteritis and IL-10^−/−^ mice, we wanted to check for its effects on MC from murine origin. Thus, bone marrow-derived MC (BMMC) were incubated either with a concentration of 50 µM resveratrol prior to IgE-dependent stimulation or lipopolysaccharide (LPS) stimulation. Degranulation, measured as β-hexosaminidase release, was almost completely inhibited by resveratrol treatment in IgE-dependently activated BMMC (Figure 4A). Moreover, in IgE-activated BMMC mRNA expression of pro-inflammatory cytokines CCL2 (Figure 4B) and TNF-α (Figure 4C) were inhibited to the level of unstimulated controls. In addition, we observed strongly reduced mRNA expression of the cytokine TNF-α in response to treatment with resveratrol prior to stimulation with LPS (Figure 4D). 

## 3. Discussion

In this study we could demonstrate that OVA-induced allergic Th2 immune response results in a strong increase in MC numbers in the GIT and that resveratrol treatment inhibits this increase in MC as well as the increased expression of the α-chain of the IL-3 receptor, the murine MC growth factor. We also found an increase in MC in experimental IL-10^−/−^ colitis compared to wildtype mice, which, again, was diminished by resveratrol treatment. Together with our findings in vitro showing a strong inhibitory effect of resveratrol on MC activation, these data indicate that resveratrol may be considered as nutraceutical in the treatment of MC associated diseases such as allergies or IBD. 

Histological changes observed in allergen-induced enteritis are combined with the infiltration of inflammatory cells into different layers of the intestinal wall [28,48,49]. We observed pronounced MC numbers in duodenum and colon of OVA-challenged BALB/c mice. This is in concordance with observations from Brandt et al. [28], who showed increased MC numbers in OVA-treated mice and that diarrhea was mediated by MC presence. Increased numbers of MC in affected tissue sections are reported by several studies examining food allergy [59,60] together with elevated levels of MC associated parameters such as MC protease 1 (MCPT1) [24,48,60] or histamine [24,57,61] in the respective intestinal areas or sera. We found that resveratrol treatment for 28 days inhibited the increased MC presence in both GIT sections duodenum and colon. In accordance with our data, resveratrol was shown to be able to prevent passive cutaneous anaphylaxis in mice [62] as well as onset of food allergy [47]. 

IL-3 is known to be the major growth factor for murine MC [58,63]. Moreover, binding of IL-3 to its receptor induces release of several cytokines such as CXCL8 [64]. IL-3R is also expressed in hiMC and hiMC growth is enhanced if cells were cultured with stem cell factor (SCF) together with IL-3. Enhanced histamine as well as leukotriene C4 (LTC4) release after FcεRI-crosslinking could also be detected [65]. We observed the enhancement of IL-3 receptor α chain (IL-3Rα) expression in OVA enteritis. This finding is in accordance with the increased MC numbers in OVA enteritis. Noteworthy, the increased expression of IL-3Rα was totally blocked by resveratrol. This finding strongly supports the assumption that MC numbers are regulated by the expression of the growth factor receptor and that resveratrol limits MC numbers by blocking IL-3Rα expression. 

Elevated presence of MC may be accompanied with enhanced MC activity. The enhancement of proteases levels in food allergy was previously shown [48,57,66]. However, we did not find significant inhibitory effects of resveratrol on mRNA expression of MC proteases in duodenum and colon sections, which have been strongly enhanced in OVA-challenged animals. 

OVA is a common allergen to induce several experimental allergic reactions in mice [45,46,47,48,59,67], which can be accompanied by clinical symptoms such as weight loss in affected animals [31,48,68]. However, we did not observe significant effects on weight changes in OVA-treated mice compared to control mice (Figure S5). In mouse models of atopic dermatitis evaluating the role of resveratrol, differences in body weight gain also did not differ between the study groups [69,70]. Onset of diarrhea is one of the most described clinical symptoms in food allergy [28]. We observed stool softening after the 4th allergen challenge, but none of the animals showed signs of severe diarrhea throughout the whole experiment. Brandt and colleagues [28] described diarrhea after the 3rd and 4th OVA applications. From a total of 10 allergen challenges, Huang et al. observed diarrhea symptoms not before the 6th one [71,72]. In a study examining the role of coumarin in OVA anaphylaxis, diarrhea score increased with ongoing allergen challenges [66]. Thus, the absence of severe diarrhea in our experiments could be the result of the shorter treatment time and severe diarrhea may be appeared with ongoing OVA challenges and elongated experiment time. 

Besides infiltration with inflammatory cells into affected tissues, food allergies are further associated with several histological changes in the intestine [48,55,56,57]. We observed that tissue damage, evaluated as epithelial barrier disruption, was slightly enhanced in intestinal tissues of OVA mice, with no significant attenuation by resveratrol. In contrast to OVA-induced enteritis, changes on histological levels were clearly detectable in IL-10^−/−^ mice which develop a spontaneous form of chronic colitis due to the missing anti-inflammatory cytokine IL-10 [53]. We observed significantly increased epithelial damage, a reduced number of goblet cells and immune cell infiltration in all IL-10^−/−^ groups as well as delayed onset of colitis symptoms. Treatment with resveratrol resulted in a down-regulation of these scores to levels not different from those of control wildtype animals. Moreover, resveratrol was also able to inhibit the increase in MC numbers in duodenum and colon tissue of colitis mice as found for MC numbers in OVA enteritis.

Although we observed strong inhibitory effects of resveratrol on the increase in MC counts, the effects on MC mediator release were less clear. Recently, we reported that resveratrol is a very potent inhibitor of hiMC. Pre-treatment with resveratrol almost abolished degranulation and expression of the cytokines CXCL8, CCL2, CCL3, CCL4 and TNF-α in hiMC in response to FcεRI receptor crosslinking [40]. Here, we examined the anti-inflammatory and anti-allergic effect of resveratrol on BMMC generated from wildtype mice. As found for human MC, resveratrol significantly diminished β-hexosaminidase release as well as gene expression of *Tnf-α* and *Ccl2* after IgE-mediated activation of BMMC. Similar observations of inhibitory effects of resveratrol on IgE-dependently activated MC were previously reported [39,73]. β-hexosaminidase or histamine release by MC may be affected by several signaling pathways initiated via FcεRI crosslinking. For example, studies already showed that resveratrol inhibits type II phosphatidylinositol (PtdIns) 4 kinase [74], phosphorylation of protein kinase C-µ (PKC-µ), phospholipase-γ (PLC-γ) and spleen tyrosine kinase Syk [62]. Additionally, ATP generated from oxidative phosphorylation in mitochondria was already shown to play an important role for MC degranulation [75] and that activated mitochondrial STAT3 as well as mitochondrial ERK1/2 are blocked by resveratrol [40]. 

In case of chronic inflammation and bacterial infections, the cell wall component of Gram-negative bacteria LPS is bound to CD14 membrane protein necessary for activation of MC via Toll-like receptor 4 (TLR4) which initiates cytokine and chemokine production in MC [76,77]. We were able to show that treatment with resveratrol totally blocked the mRNA expression of the pro-inflammatory cytokine TNF-α in both BMMC treated with LPS and BMMC stimulated by FcεRI receptor crosslinking. Li et al. [73] detected decreased TNF-α and IL-6 secretion in IgE mediated activated BMMC in response to a concentration of 10 µM resveratrol. Release of IL-6, TNF-α and IL-13 was reduced by more than 50% by a concentration of 25 µM resveratrol in IgE-activated BMMC [21]. We found a complete inhibition of IgE-mediated mRNA expression of Tnf-α and Ccl2 in response to treatment with 50 µM resveratrol. CCL2 and TNF-α serve as regulatory factors in endothelial and immune regulation by attracting macrophages, neutrophils and basophils to inflammation sites and therefore promoting inflammatory reactions [78,79,80]. TNF-α as well as CCL2 are also relevant in pathogenesis of IBD as they are released by MC during early stages of inflammation and needed for sustaining colitis [81]. Inhibition of CCL2 pathway was shown to prevent a Th2 response in allergic asthma [82]. 

Not only resveratrol but also several other natural plant substances have been shown to have an anti-inflammatory effect on MC. In in vivo OVA models these were, amongst others, Chinese sweet tea (1% weight/volume (*w*/*v*)) [61], the flavonoid dihydromyricetin (10 mg/kg) [83], polyphenols from Arecae semen (0.1% *w*/*v*) [60] or the polyphenol fisetin (3 mg/kg/day) [84]. Degranulation of MC in jejuni (ca. 50%) together with reduction in MCPT1 (ca. 15%) and histamine levels (ca. 60%) of mice suffering from Th2-induced food allergy was further attenuated by polysaccharides from Aloe vera gel (100 mg/kg) [49]. We found that citrus flavonoids tangeretin and especially nobiletin affect activation of hiMC [85]. Moreover, we found that cinnamon extract, similar to resveratrol, was a more potent inhibitor of MC activation than the citrus flavonoids [40,51,52]. On the other side, cinnamon extract and nobiletin showed clearer effects on the attenuation of symptoms in the pathology of colitis in IL-10^−/−^ mice than resveratrol [51,52,86]; nonetheless, similar to resveratrol, nobiletin also delayed the onset of symptoms in affected animals during the experimental course [52]. These observations lead to the assumption that nobiletin and cinnamon extract may be more auspicious substances than resveratrol in the treatment of colitis [51,52,87]. This may be due to the low bioavailability of resveratrol [38,88] which should be in the focus of future research. The poor bioavailability of resveratrol is due to its transformation into glucuronide and sulphate derivatives, both in liver and intestine [38]. It is important to note that most of the resveratrol is excreted unmetabolized (75%) and that the highest detected amount of free resveratrol was 1.7–1.9% [88]. Even though bioavailability of resveratrol or other polyphenols [89] seems to be a major problem in using them as nutraceutical, there are numerous studies reporting anti-inflammatory effects of resveratrol when applied either via oral gavage [24,46], chow [45,47] or as additive in drinking water [90,91]. Nevertheless, it seems to be necessary to increase the bioavailability of polyphenols, e.g., by encapsulation with carrier substances [46,92,93]. Regarding the application of resveratrol in humans, trials using concentration ranging from 10 mg to 5 g have already been carried out successfully [94,95,96]. Nonetheless, concentrations higher than 500 mg provoked mild to moderate gastrointestinal symptoms [96,97]. A concentration of 50 mg/kg BW in mice corresponds a dose of 243–324 mg in adult humans weighting 60–80 kg [98,99], respectively, and these concentrations are in the range of the concentrations commonly used for human trials and stated to be safe [96,97]. In addition, these levels are below the highest commercially available single dose of about 500 mg per tablet or capsule [100]. In vitro concentrations of resveratrol are also varying from low of 0.03 µM [20] to high doses of 100–200 µM [19], depending on the in vitro models used. The high metabolism and excretion of resveratrol [87] leads to low physiological concentrations (50 nM–2 µM), so that the in vitro concentrations used cannot be reached physiologically by the consumption of resveratrol in food [93]. 

In conclusion, our results demonstrate that resveratrol treatment prevents the increase in MC in allergen-induced Th2 enteritis as well as in experimental IL-10^−/−^ colitis. Inhibitory effects of resveratrol on MC regarding degranulation and cytokine expression were also found in vitro. Therefore, resveratrol may be considered as an anti-allergic and anti-inflammatory plant-derived component for the prevention or treatment of MC-associated disorders.

## 4. Materials and Methods

### 4.1. Animals and Treatments

Four-week-old male BALB/c wild type mice (Janvier Labs, Le Genest-Saint-Isle, France) or IL-10 knockout mice (IL-10^−/−^) with a BALB/c background were kept in a specific pathogen-free barrier (SPF) facility under controlled conditions and a light/dark cycle of 12 h accredited by the Association for Assessment and Accreditation of Laboratory Animal Care. Mice were kept one mouse per cage and were provided a standard diet (ssniff Spezialdiäten GmbH, Soest, Germany) and drinking water ad libitum. All treatments and procedures were approved by the local Institutional Animal Care and Use Committee of the Ministry of Agriculture, Rural Areas, Veterinary and Food Sector of Stuttgart (permission number: V364/20; RPS35-9185.99/363, permission received on 24th June 2020). OVA enteritis model was as follows: mice were randomly divided into six groups (each n = 8), three for control and three for OVA treatment, receiving either no additive, 50 mg/kg body weight/d ethanol (≥99.8%; Carl Roth, Karlsruhe, Germany) or 50 mg/kg body weight/day resveratrol (dissolved in ≥99.8% ethanol; Carl Roth) in drinking water. Animals of the OVA groups were intraperitoneally (i.p.) sensitized with 50 µg OVA (Genaxxon bioscience, Ulm, Germany) in alum (1:2) (Thermo Fisher Scientific, Bonn, Germany) on challenge day 5 and 11. Oral gavage of 50 mg OVA dissolved in 250 µL 0.9% physiologic salt solution (NaCl; B. Braun, Melsungen, Germany) was carried out 6 times on challenge day 15, 18, 20, 22, 25, and 27. IL-10^−/−^ colitis model: Mice were randomly divided into six groups, three groups consisting of BALB/c wildtype mice (each n = 5) and three groups of IL-10^−/−^ mice (each n = 10). Thereby, animals received either no additive, 50 mg/kg body weight/day ethanol or 50 mg/kg body weight/day resveratrol (dissolved in ethanol) in drinking water for a total time span of 90 days. Drinking volume and body weight were determined every second day and quantity of ethanol or resveratrol was adjusted to the drinking volume of every mouse. Mice were anesthetized with 100 mg ketamine/kg body weight and 16 mg xylazine/kg body weight i.p. and sacrificed by cervical dislocation on experimental day 28 (OVA model) or day 90 (IL-10^−/−^) when showing no previous signs of inflammation.

### 4.2. Assessment of OVA-Induced Allergic Enteritis and Colitis in IL-10^−/−^ Mice

Severity of enteritis and colitis was monitored everyday based on a scoring system, accredited by the local ethics committee, with scores ranging from 0 to 3. Important end points of this scoring system were amongst others body weight change, as follows: 0: 0–1%, 1: 1–10%, 2: 10–20%, 3: ≥20%; rectal inflammation: 3: prolapse and/or rectal bleeding and consistency of stool: 3: diarrhea. Scoring of the stool followed the respective criteria, as follows: 0: normal, firm and round-formed feces; 1: soft and round-formed feces; 2: very soft and in parts unformed feces; 3: diarrhea. Mice were killed after reaching a single score of 2. When reaching a cumulative score of 3 or more, mice were killed after a 24 h observation period without recovery. Tissue samples of small intestine and colon were immediately fixed in 4% phosphate buffered formalin solution (Carl Roth) for later embedding or frozen immediately in liquid nitrogen for later isolation of RNA.

Intestinal inflammation was examined using formalin-fixed and paraffin-embedded (Carl Roth) samples of duodenum and colon stained with hematoxylin (Sigma Aldrich, Darmstadt, Germany) and eosin (Sigma Aldrich) (H&E) as previously described [51]. The scores ranged from 0 to 3 and contained the criteria for tissue injury (score 0: undamaged mucosa, 1: single lymphoepithelial damages, 2: surface damages of mucosa, 3: extensive mucosal damage and damage of deeper structures of the intestinal wall), number of goblet cells (score 0: normal; score 1: <50% reduction; score 2: 50–90% reduction; score 3: >90% reduction), and infiltration of inflammatory cells (score 0: low numbers of inflammatory cells in lamina propria; score 1: increased number of inflammatory cells in the lamina propria; score 2: accumulation of inflammatory cells in the lamina propria and infiltration into the submucosa; 3: transmural distribution of inflammatory cells) (Figure S6). Further, bowel wall thickness was measured from muscularis externa to crypt base.

### 4.3. Histological Analysis of MC

Formalin-fixed tissue samples were embedded in paraffin. After deparaffinization and rehydration, 5 µM thick sections were stained with toluidine blue (Carl Roth) for visualization of MC as previously described [51,52]. Total MC number was obtained at 200–400x in the high field. Microscopic visualization of all parameters was conducted by usage of AxioVision software (Carl Zeiss Microscopy, Jena, Germany).

### 4.4. Generation of BMMC

Skin and muscles were removed from tibia and femur of wild type mice and DPBS (Gibco; Thermo Fisher Scientific) was used for rinsing out the bone marrow cells. Cells were counted and suspended in 90% fetal calf serum (FCS; Merck; Darmstadt, Germany) with 10% DMSO (Carl Roth) and frozen in liquid nitrogen until further processing. Bone marrow cells were cultured in an overall volume of 5 mL BMMC medium (RPMI1640 GlutaMaxTM (Gibco; Thermo Fisher Scientific) with 10% FCS, 1% penicillin-streptomycin solution (HyCloneTM Laboratories, South Logan, USA) in the presence of murine IL-3 with a final concentration of 30 ng/mL (Peprotech, Hamburg, Germany). During the first 5 weeks of cultivation, medium and plates were changed twice a week, then medium was changed once a week to remove adherent cells. Suspension cells increased in size and developed a round shape. After culturing for 6 to 8 weeks the cells were used for functional assays. Maturity and purity of the BMMC were examined on cytospins stained with May-Grünwald/Giemsa (Carl Roth, medite histotechnic, Burgdorf, Germany).

### 4.5. Treatment of BMMC

Cells were treated with 50 µM resveratrol (Sigma Aldrich, St. Louis, MO, USA) 1 h prior to incubation with IgE-specific 2,4-dinitrophenol (IgE-DNP; provided by U. Blank, French Institute of Health and Medical Research, Paris, France) for 90 min at 37 °C. Cells were washed twice with DPBS and stimulated with 10 µg/mL DNP (Thermo Fisher Scientific) for 90 min at 37 °C to analyze mRNA expression or 30 min to determine β-hexosaminidase release. For LPS stimulation, cells were treated with 50 µM resveratrol for 1 h prior to 1 µg/mL LPS (Escherichia coli O111:B4; Sigma Aldrich) stimulation for 3 h. Unstimulated controls contained the same concentrations of the vehicle DMSO.

### 4.6. Measurement of Degranulation 

Degranulation of BMMC was measured by determining the amount of released β-hexosaminidase in supernatants by a color enzyme assay [101]. In brief, cell supernatants were incubated with 50 µL of 4-nitrophenyl-*N*-acetyl-β-d-glucosamid (pNAG; Carl Roth) for 1h hour at 37 °C. The enzymatic conversion of pNAG by β-hexosaminidase into 4-nitrophenol was stopped with 150 µL of 0.2 glycine (pH 10.7; Carl Roth). β-hexosaminidase release was measured by its enzymatic 4-nitrophenol product in a photometer at 405 nm wavelength.

### 4.7. RNA Preparation and Real-Time RT-PCR

Total RNA was obtained from cell lysates using EXTRACTME^®^ TOTAL RNA kit (blirt, Gdansk, Poland) and from tissue samples using peqGold TrifastTM (VWR International GmbH, Erlangen, Germany). Real-time RT-PCR reactions were performed in optical tubes containing cDNA template, each sense and anti-sense primer, and SsoFastTM EVAGreen Supermix (Bio-Rad Laboratories, Feldkirchen, Germany). Reaction mixture without cDNA was used as negative control. Relative quantification (2^−ΔΔCT^) was performed using glyceraldehyde 3-phosphate dehydrogenase (*Gapdh*) housekeeping gene as reference. Mouse sense and antisense primer sequences were as follows: *Gapdh*: 5′-TGT TCC TAC CCC CAA TGT GT-3′, 5′-AGA GTG GGA GTT GCT GTT GA-3′, product size: 175 bp; *Ccl2*: 5′-ACT CAC CTG CTG CTA CTC AT-3′, 5′-TCA GCA CAG ACC TCT CTC TT-3′, product size: 138 bp; *Il-3rα*: 5′-TGG AGG AAG TCG CTG CTC TA-3′, 5′-CGT CAC CTC GCA GTC TTC AA-3′, product size: 111 bp; *Tnf-α*: 5′-ACC ACC ATC AAG GAC TCA-3′, 5′-AGG TCT GAA GGT AGG AAG G-3′, product size: 127 bp; *Mc-cpa*: 5′-CAT GGA CAC AGG ATC GAA TG-3′, 5′-TGC AGG TCC CCT GTA GAC AT-3′, product size: 152 bp; *Mcpt4*: 5′-ATC TTA TGG ACG CGG AGA TG-3′, 5′-GTG ACA GGA TGG ACA CAT GC-3′, product size: 185 bp; (all Eurofins Genomics, Ebersberg, Germany). The CFX 2.1 software and CFX Connect Real-Time PCR System of Bio-Rad Laboratories were used.

### 4.8. Statistics

Data are expressed as mean ± standard error of the mean (SEM). If not stated otherwise, student’s *t*-test was used for differences in in vitro experiments and one-way analysis of variance (ANOVA) with Tukey’s post hoc test was used to analyze differences between treatment groups in in vivo experiments. Statistically significant differences between treatment groups are shown by different letters. Common letters between treatment groups mean no significant difference. A value of *p* < 0.05 is considered to be statistically significant. GraphPad Prism scientific software version 5.0 (San Diego, CA, USA) was used for statistical analysis.

## Data Availability

Data are available on request from the authors.

## Supplementary Materials

The following supporting information can be downloaded at: https://www.mdpi.com/article/10.3390/ijms23031213/s1.

## References

[1] Gupta R.S., Warren C.M., Smith B.M., Jiang J., Blumenstock J.A., Davis M.M., Schleimer R.P., Nadeau K.C. (2019). Prevalence and Severity of Food Allergies Among US Adults. JAMA Netw. Open.

[2] Kaplan G.G., Ng S.C. (2017). Understanding and Preventing the Global Increase of Inflammatory Bowel Disease. Gastroenterology.

[3] Volmer T., Effenberger T., Trautner C., Buhl R. (2018). Consequences of long-term oral corticosteroid therapy and its side-effects in severe asthma in adults: A focused review of the impact data in the literature. Eur. Respir. J..

[4] Rogler G. (2010). Gastrointestinal and liver adverse effects of drugs used for treating IBD. Best Pr. Res. Clin. Gastroenterol..

[5] Kornbluth A., Sachar D.B., Practice Parameters Committee of the American College of Gastroenterology (2010). Ulcerative Colitis Practice Guidelines in Adults: American College of Gastroenterology, Practice Parameters Committee. Am. J. Gastroenterol..

[6] Baumgart D.C., Sandborn W.J. (2007). Inflammatory bowel disease: Clinical aspects and established and evolving therapies. Lancet.

[7] Baiardini I., Novakova S., Mihaicuta S., Oguzulgen I.K., Canonica G.W. (2018). Adherence to treatment in allergic respiratory diseases. Expert Rev. Respir. Med..

[8] Lemberg M.-L., Joisten M.-J., Mösges R. (2017). Adhärenz in der spezifischen Immuntherapie Adherence in specific immunotherapy. Der Hautarzt.

[9] Kiel M.A., Röder E., van Wijk R.G., Al M.J., Hop W.C., Molken M.R.-V. (2013). Real-life compliance and persistence among users of subcutaneous and sublingual allergen immunotherapy. J. Allergy Clin. Immunol..

[10] Souverein P.C., Koster E.S., Colice G., van Ganse E., Chisholm A., Price D., Dima A.L., Respiratory Effectiveness Group’s Adherence Working Group (2017). Inhaled Corticosteroid Adherence Patterns in a Longitudinal Asthma Cohort. J. Allergy Clin. Immunol. Pr..

[11] Ganesh V., Banigo A., McMurran A.E.L., Shakeel M., Ram B. (2017). Does intranasal steroid spray technique affect side effects and compliance? Results of a patient survey. J. Laryngol. Otol..

[12] Wang T., Li Y., Wang F., Zhou C. (2017). Nonadherence to sublingual immunotherapy in allergic rhinitis: A real-life analysis. Int. Forum Allergy Rhinol..

[13] Khan N., Abbas A.M., Bazzano L.A., Koleva Y.N., Krousel-Wood M. (2012). Long-term oral mesalazine adherence and the risk of disease flare in ulcerative colitis: Nationwide 10-year retrospective cohort from the veterans affairs healthcare system. Aliment. Pharmacol. Ther..

[14] Kucuksezer U.C., Ozdemir C., Cevhertas L., Ogulur I., Akdis M., Akdis C.A. (2020). Mechanisms of allergen-specific immunotherapy and allergen tolerance. Allergol. Int..

[15] Shakoor H., Feehan J., Apostolopoulos V., Platat C., Al Dhaheri A., Ali H., Ismail L., Bosevski M., Stojanovska L. (2021). Immunomodulatory Effects of Dietary Polyphenols. Nutrients.

[16] Maleki S.J., Crespo J.F., Cabanillas B. (2019). Anti-inflammatory effects of flavonoids. Food Chem..

[17] Malaguarnera L. (2019). Influence of Resveratrol on the Immune Response. Nutrients.

[18] Redegeld F.A., Yu Y., Kumari S., Charles N., Blank U. (2018). Non-IgE mediated mast cell activation. Immunol. Rev..

[19] Wang J., Zhang Y., Hu S., Ge S., Jia M., Wang N. (2021). Resveratrol inhibits MRGPRX2-mediated mast cell activation via Nrf2 pathway. Int. Immunopharmacol..

[20] Moon P.-D., Han N.-R., Lee J., Jee H.-W., Kim J.-H., Kim H.-M., Jeong H.-J. (2020). Effects of Resveratrol on Thymic Stromal Lymphopoietin Expression in Mast Cells. Medicina.

[21] Nakajima S., Ishimaru K., Kobayashi A., Yu G., Nakamura Y., Oh-Oka K., Suzuki-Inoue K., Kono K., Nakao A. (2019). Resveratrol inhibits IL-33–mediated mast cell activation by targeting the MK2/3–PI3K/Akt axis. Sci. Rep..

[22] Xu Y., Liu Q., Guo X., Xiang L., Zhao G. (2020). Resveratrol attenuates IL-33-induced mast cell inflammation associated with inhibition of NF-κB activation and the P38 signaling pathway. Mol. Med. Rep..

[23] Wang J., Chen G., Lu L., Zou H. (2019). Sirt1 inhibits gouty arthritis via activating PPARγ. Clin. Rheumatol..

[24] Zhang Y.-F., Liu Q.-M., Gao Y.-Y., Liu B., Liu H., Cao M.-J., Yang X.-W., Liu G.-M. (2019). Attenuation of allergic responses following treatment with resveratrol in anaphylactic models and IgE-mediated mast cells. Food Funct..

[25] Lv C., Zhang Y., Shen L. (2018). Preliminary Clinical Effect Evaluation of Resveratrol in Adults with Allergic Rhinitis. Int. Arch. Allergy Immunol..

[26] del Giudice M.M., Maiello N., Capristo C., Alterio E., Capasso M., Perrone L., Ciprandi G. (2014). Resveratrol plus carboxymethyl-β-glucan reduces nasal symptoms in children with pollen-induced allergic rhinitis. Curr. Med. Res. Opin..

[27] Fricker M., Qin L., Niessen N., Baines K., McDonald V.M., Scott H.A., Simpson J.L., Gibson P.G. (2020). Relationship of sputum mast cells with clinical and inflammatory characteristics of asthma. Clin. Exp. Allergy.

[28] Brandt E., Strait R.T., Hershko D., Wang Q., Muntel E.E., Scribner T.A., Zimmermann N., Finkelman F.D., Rothenberg M.E. (2003). Mast cells are required for experimental oral allergen–induced diarrhea. J. Clin. Investig..

[29] Lorentz A., Schwengberg S., Mierke C., Manns M.P., Bischoff S.C. (1999). Human intestinal mast cells produce IL-5 in vitro upon IgE receptor cross-linking and in vivo in the course of intestinal inflammatory disease. Eur. J. Immunol..

[30] Chichlowski M., Westwood G.S., Abraham S.N., Hale L.P. (2010). Role of Mast Cells in Inflammatory Bowel Disease and Inflammation-Associated Colorectal Neoplasia in IL-10-Deficient Mice. PLoS ONE.

[31] Rijnierse A., Nijkamp F.P., Kraneveld A.D. (2007). Mast cells and nerves tickle in the tummy: Implications for inflammatory bowel disease and irritable bowel syndrome. Pharmacol. Ther..

[32] Bischoff S.C. (2016). Mast cells in gastrointestinal disorders. Eur. J. Pharmacol..

[33] Hamilton M.J., Sinnamon M.J., Lyng G.D., Glickman J.N., Wang X., Xing W., Krilis S.A., Blumberg R.S., Adachi R., Lee D.M. (2010). Essential role for mast cell tryptase in acute experimental colitis. Proc. Natl. Acad. Sci. USA.

[34] Boeckxstaens G. (2015). Mast cells and inflammatory bowel disease. Curr. Opin. Pharmacol..

[35] Hansbro P.M., Hamilton M.J., Fricker M., Gellatly S.L., Jarnicki A.G., Zheng D., Foster P.S. (2014). Importance of mast cell Prss31/transmembrane tryptase/tryptase-γ in lung function and experimental chronic obstructive pulmonary disease and colitis. J. Biol. Chem..

[36] Ahn J.Y., Lee K.H., Choi C.H., Kim J.W., Lee H.W., Kim J.W., Kim M.K., Kwon G.Y., Han S., Kim S.-E. (2013). Colonic Mucosal Immune Activity in Irritable Bowel Syndrome: Comparison with Healthy Controls and Patients with Ulcerative Colitis. Dig. Dis. Sci..

[37] Bedmar M.T.C., Heil S.D.S., Myrelid P., Söderholm J.D., Keita Å.V. (2018). Upregulation of intestinal mucosal mast cells expressing VPAC1 in close proximity to vasoactive intestinal polypeptide in inflammatory bowel disease and murine colitis. Neurogastroenterol. Motil..

[38] Perrone D., Fuggetta M.P., Ardito F., Cottarelli A., de Filippis A., Ravagnan G., de Maria S., Muzio L.L. (2017). Resveratrol (3,5,4′-trihydroxystilbene) and its properties in oral diseases. Exp. Ther. Med..

[39] de Sá Coutinho D., Pacheco M.T., Frozza R.L., Bernardi A. (2018). Anti-Inflammatory Effects of Resveratrol: Mechanistic Insights. Int. J. Mol. Sci..

[40] Bilotta S., Paruchuru L., Feilhauer K., Köninger J., Lorentz A. (2021). Resveratrol Is a Natural Inhibitor of Human Intestinal Mast Cell Activation and Phosphorylation of Mitochondrial ERK1/2 and STAT3. Int. J. Mol. Sci..

[41] Royce S.G., Dang W., Yuan G., Tran J., El Osta A., Karagiannis T.C., Tang M.L. (2011). Resveratrol has protective effects against airway remodeling and airway hyperreactivity in a murine model of allergic airways disease. Pathobiol. Aging Age-Relat. Dis..

[42] Lee H.Y., Kim I.K., Yoon H.K., Kwon S., Rhee C.K., Lee S.Y. (2017). Inhibitory Effects of Resveratrol on Airway Remodeling by Transforming Growth Factor-β/Smad Signaling Pathway in Chronic Asthma Model. Allergy Asthma Immunol. Res..

[43] Shen Y., Xu J. (2019). Resveratrol Exerts Therapeutic Effects on Mice With Atopic Dermatitis. Wounds.

[44] Zhang W., Tang R., Ba G., Li M., Lin H. (2020). Anti-allergic and anti-inflammatory effects of resveratrol via inhibiting TXNIP-oxidative stress pathway in a mouse model of allergic rhinitis. World Allergy Organ. J..

[45] Li J., Wang B., Luo Y., Zhang Q., Bian Y., Wang R. (2020). Resveratrol-mediated SIRT1 activation attenuates ovalbumin-induced allergic rhinitis in mice. Mol. Immunol..

[46] Alharris E., Alghetaa H., Seth R., Chatterjee S., Singh N.P., Nagarkatti M., Nagarkatti P. (2018). Resveratrol Attenuates Allergic Asthma and Associated Inflammation in the Lungs Through Regulation of miRNA-34a That Targets FoxP3 in Mice. Front. Immunol..

[47] Okada Y., Oh-Oka K., Nakamura Y., Ishimaru K., Matsuoka S., Okumura K., Ogawa H., Hisamoto M., Okuda T., Nakao A. (2012). Dietary Resveratrol Prevents the Development of Food Allergy in Mice. PLoS ONE.

[48] Blanco-Pérez F., Kato Y., Gonzalez-Menendez I., Laiño J., Ohbayashi M., Burggraf M., Krause M., Kirberg J., Iwakura Y., Martella M. (2019). CCR8 leads to eosinophil migration and regulates neutrophil migration in murine allergic enteritis. Sci. Rep..

[49] Lee D., Kim H.S., Shin E., Do S.-G., Lee C.-K., Kim Y.M., Lee M.B., Min K.Y., Koo J., Kim S.J. (2018). Polysaccharide isolated from Aloe vera gel suppresses ovalbumin-induced food allergy through inhibition of Th2 immunity in mice. Biomed. Pharmacother..

[50] Lennon E.M., Borst L., Edwards L.L., Moeser A.J. (2018). Mast Cells Exert Anti-Inflammatory Effects in an IL10−/−Model of Spontaneous Colitis. Mediat. Inflamm..

[51] Hagenlocher Y., Hösel A., Bischoff S.C., Lorentz A. (2016). Cinnamon extract reduces symptoms, inflammatory mediators and mast cell markers in murine IL-10−/− colitis. J. Nutr. Biochem..

[52] Hagenlocher Y., Gommeringer S., Held A., Feilhauer K., Köninger J., Bischoff S.C., Lorentz A. (2018). Nobiletin acts anti-inflammatory on murine IL-10−/− colitis and human intestinal fibroblasts. Eur. J. Nutr..

[53] Kühn R., Löhler J., Rennick D.M., Rajewsky K., Muller W. (1993). Interleukin-10-deficient mice develop chronic enterocolitis. Cell.

[54] Martorell A., Alonso E., Boné J., Echeverría L., López M., Martín F., Nevot S., Plaza A. (2013). Position document: IgE-mediated allergy to egg protein. Allergol. Immunopathol..

[55] Saldanha J.C.S., Gargiulo D.L., Silva S.S., Carmo-Pinto F.H., Andrade M.C., Alvarez-Leite J.I., Teixeira M.M., Cara D.C. (2004). A model of chronic IgE-mediated food allergy in ovalbumin-sensitized mice. Braz. J. Med Biol. Res..

[56] Cardoso C.R.D.B., Provinciatto P.R., Godoi D.F., Ferreira B.R., Teixeira G., Rossi M.A., Cunha F.Q., Silva J.S. (2009). IL-4 regulates susceptibility to intestinal inflammation in murine food allergy. Am. J. Physiol. Liver Physiol..

[57] Reyes-Pavón D., Cervantes-García D., Bermúdez-Humarán L.G., Córdova-Dávalos L.E., Quintanar-Stephano A., Jiménez M., Salinas E. (2020). Protective Effect of Glycomacropeptide on Food Allergy with Gastrointestinal Manifestations in a Rat Model through Down-Regulation of Type 2 Immune Response. Nutrients.

[58] Lantz C.S., Boesiger J., Song C.H., Mach N., Kobayashi T., Mulligan R.C., Nawa Y., Dranoff G., Galli S.J. (1998). Role for interleukin-3 in mast-cell and basophil development and in immunity to parasites. Nature.

[59] Burggraf M., Nakajima-Adachi H., Hachimura S., Ilchmann A., Pemberton A.D., Kiyono H., Vieths S., Toda M. (2011). Oral tolerance induction does not resolve gastrointestinal inflammation in a mouse model offoodallergy. Mol. Nutr. Food Res..

[60] Wang C.-C., Lin Y.-R., Liao M.-H., Jan T.-R. (2013). Oral supplementation with areca-derived polyphenols attenuates food allergic responses in ovalbumin-sensitized mice. BMC Complement. Altern. Med..

[61] Mine Y., Majumder K., Jin Y., Zeng Y. (2020). Chinese sweet tea (*Rubus suavissimus*) polyphenols attenuate the allergic responses in a Balb/c mouse model of egg allergy. J. Funct. Foods.

[62] Han S.-Y., Bae J.-Y., Park S.-H., Kim Y.-H., Park J.H.Y., Kang Y.-H. (2013). Resveratrol Inhibits IgE-Mediated Basophilic Mast Cell Degranulation and Passive Cutaneous Anaphylaxis in Mice. J. Nutr..

[63] Rottem M., Hull G., Metcalfe D.D. (1994). Demonstration of differential effects of cytokines on mast cells derived from murine bone marrow and peripheral blood mononuclear cells. Exp. Hematol..

[64] Varricchi G., Poto R., Marone G., Schroeder J.T. (2021). IL-3 in the development and function of basophils. Semin. Immunol..

[65] Gebhardt T., Sellge G., Lorentz A., Raab R., Manns M.P., Bischoff S.C. (2002). Cultured human intestinal mast cells express func-tional IL-3 receptors and respond to IL-3 by enhancing growth and IgE receptor-dependent mediator release. Eur. J. Immunol..

[66] Liu Q.-M., Zhang Y.-F., Gao Y.-Y., Liu H., Cao M.-J., Yang X.-W., Su W.-J., Liu G.-M. (2019). Coumarin alleviates ovalbumin-induced food anaphylaxis in a mouse model by affecting mast cell function. Food Funct..

[67] Kumar R.K., Herbert C., Foster P.S. (2008). The “Classical” Ovalbumin Challenge Model of Asthma in Mice. Curr. Drug Targets.

[68] Nakajima-Adachi H., Ebihara A., Kikuchi A., Ishida T., Sasaki K., Hirano K., Watanabe H., Asai K., Takahashi Y., Kanamori Y. (2006). Food antigen causes TH2-dependent enteropathy followed by tissue repair in T-cell receptor transgenic mice. J. Allergy Clin. Immunol..

[69] Gounder V.K., Arumugam S., Thandavarayan R.A., Pitchaimani V., Sreedhar R., Afrin R., Harima M., Suzuki H., Nomoto M., Miyashita S. (2014). Resveratrol attenuates HMGB1 signaling and inflammation in house dust mite-induced atopic dermatitis in mice. Int. Immunopharmacol..

[70] Sozmen S.C., Karaman M., Micili S.C., Isik S., Ayyildiz Z.A., Bağrıyanık H.A., Uzuner N., Karaman O. (2016). Resveratrol ameliorates 2,4-dinitrofluorobenzene-induced atopic dermatitis-like lesions through effects on the epithelium. PeerJ.

[71] Huang C.-H., Ku C.-Y., Jan T.-R. (2009). Diosgenin Attenuates Allergen-Induced Intestinal Inflammation and IgE Production in a Murine Model of Food Allergy. Planta Med..

[72] Huang C.-H., Pan C.-L., Tsai G.-J., Chang C.-J., Tsai W.-C., Lu S.-Y. (2021). Anti-Allergic Diarrhea Effect of Diosgenin Occurs via Improving Gut Dysbiosis in a Murine Model of Food Allergy. Molecules.

[73] Li X., Lee Y.J., Jin F., Na Park Y., Deng Y., Kang Y., Yang J.H., Chang J.-H., Kim D.-Y., Kim J.-A. (2017). Sirt1 negatively regulates FcεRI-mediated mast cell activation through AMPK- and PTP1B-dependent processes. Sci. Rep..

[74] Naveen B., Shankar B., Subrahmanyam G. (2005). FcɛRI cross-linking activates a type II phosphatidylinositol 4-kinase in RBL 2H3 cells. Mol. Immunol..

[75] Erlich T.H., Yagil Z., Kay G., Peretz A., Migalovich-Sheikhet H., Tshori S., Nechushtan H., Levi-Schaffer F., Saada A., Razin E. (2014). Mitochondrial STAT3 plays a major role in IgE-antigen–mediated mast cell exocytosis. J. Allergy Clin. Immunol..

[76] McCurdy J.D., Lin T.J., Marshall J.S. (2001). Toll-like receptor 4-mediated activation of murine mast cells. J. Leukoc. Biol..

[77] Supajatura V., Ushio H., Nakao A., Okumura K., Ra C., Ogawa H. (2001). Protective Roles of Mast Cells Against Enterobacterial Infection Are Mediated by Toll-Like Receptor 4. J. Immunol..

[78] Hong L., Wang Q., Chen M., Shi J., Guo Y., Liu S., Pan R., Yuan X., Jiang S. (2021). Mas receptor activation attenuates allergic airway inflammation via inhibiting JNK/CCL2-induced macrophage recruitment. Biomed. Pharmacother..

[79] Rijnierse A., Koster A.S., Nijkamp F.P., Kraneveld A.D. (2006). TNF-α is crucial for the development of mast cell-dependent colitis in mice. Am. J. Physiol. Liver Physiol..

[80] Zhang Y., Ramos B.F., Jakschik B.A. (1992). Neutrophil Recruitment by Tumor Necrosis Factor from Mast Cells in Immune Complex Peritonitis. Science.

[81] Valeri V., Tonon S., Vibhushan S., Gulino A., Belmonte B., Adori M., Hedestam G.B.K., Gautier G., Tripodo C., Blank U. (2021). Mast cells crosstalk with B cells in the gut and sustain IgA response in the inflamed intestine. Eur. J. Immunol..

[82] Jiang S., Wang Q., Wang Y., Song X., Zhang Y. (2019). Blockade of CCL2/CCR2 signaling pathway prevents inflammatory monocyte recruitment and attenuates OVA-Induced allergic asthma in mice. Immunol. Lett..

[83] Zhang Y.-F., Liu Q.-M., Liu B., Shu Z.-D., Han J., Liu H., Cao M.-J., Yang X.-W., Guangming L., Liu G.-M. (2019). Dihydromyricetin inhibited ovalbumin-induced mice allergic responses by suppressing the activation of mast cells. Food Funct..

[84] Elkholy R., Balaha M., El-Anwar N., Kandeel S., Hedya S., Rahman M.-N.A.-E. (2018). Fisetin and telmisartan each alone or in low-dose combination alleviate OVA-induced food allergy in mice. Pharmacol. Rep..

[85] Hagenlocher Y., Feilhauer K., Schäffer M., Bischoff S.C., Lorentz A. (2017). Citrus peel polymethoxyflavones nobiletin and tangeretin suppress LPS- and IgE-mediated activation of human intestinal mast cells. Eur. J. Nutr..

[86] Hagenlocher Y., Satzinger S., Civelek M., Feilhauer K., Köninger J., Bischoff S.C., Lorentz A. (2017). Cinnamon reduces inflammatory response in intestinal fibroblasts in vitro and in colitis in vivo leading to decreased fibrosis. Mol. Nutr. Food Res..

[87] Chung M.-Y., Shin H.S., Choi D.W., Shon D.-H. (2016). Citrus Tachibana Leaf Extract Mitigates Symptoms of Food Allergy by Inhibiting Th2-Associated Responses. J. Food Sci..

[88] Pannu N., Bhatnagar A. (2019). Resveratrol: From enhanced biosynthesis and bioavailability to multitargeting chronic diseases. Biomed. Pharmacother..

[89] di Lorenzo C., Colombo F., Biella S., Stockley C., Restani P. (2021). Polyphenols and Human Health: The Role of Bioavailability. Nutrients.

[90] Andreani C., Bartolacci C., Wijnant K., Crinelli R., Bianchi M., Magnani M., Hysi A., Iezzi M., Amici A., Marchini C. (2017). Resveratrol fuels HER2 and ERα-positive breast cancer behaving as proteasome inhibitor. Aging.

[91] Campbell C.L., Yu R., Li F., Zhou Q., Chen D., Qi C., Yin Y., Sun J. (2019). Modulation of fat metabolism and gut microbiota by resveratrol on high-fat diet-induced obese mice. Diabetes Metab. Syndr. Obes. Targets Ther..

[92] Zhao J., Yang J., Xie Y. (2019). Improvement strategies for the oral bioavailability of poorly water-soluble flavonoids: An overview. Int. J. Pharm..

[93] Hu B., Liu X., Zhang C., Zeng X. (2017). Food macromolecule based nanodelivery systems for enhancing the bioavailability of polyphenols. J. Food Drug Anal..

[94] Tomé-Carneiro J., Larrosa M., González-Sarrías A., Tomas-Barberan F., Conesa M.T.G., Espín J. (2013). Resveratrol and Clinical Trials: The Crossroad from In Vitro Studies to Human Evidence. Curr. Pharm. Des..

[95] Boocock D.J., Faust G.E., Patel K.R., Schinas A.M., Brown V.A., Ducharme M.P., Booth T.D., Crowell J.A., Perloff M., Gescher A.J. (2007). Phase I Dose Escalation Pharmacokinetic Study in Healthy Volunteers of Resveratrol, a Potential Cancer Chemopreventive Agent. Cancer Epidemiol. Biomark. Prev..

[96] Brown V.A., Patel K.R., Viskaduraki M., Crowell J.A., Perloff M., Booth T.D., Vasilinin G., Sen A., Schinas A.M., Piccirilli G. (2010). Repeat Dose Study of the Cancer Chemopreventive Agent Resveratrol in Healthy Volunteers: Safety, Pharmacokinetics, and Effect on the Insulin-like Growth Factor Axis. Cancer Res..

[97] Sergides C., Chirilă M., Silvestro L., Pitta D., Pittas A. (2016). Bioavailability and safety study of resveratrol 500 mg tablets in healthy male and female volunteers. Exp. Ther. Med..

[98] Reagan-Shaw S., Nihal M., Ahmad N. (2008). Dose translation from animal to human studies revisited. FASEB J..

[99] Nair A., Morsy M., Jacob S. (2018). Dose translation between laboratory animals and human in preclinical and clinical phases of drug development. Drug Dev. Res..

[100] Dobrzyńska M.M., Gajowik A., Radzikowska J. (2016). The effect ofin vivoresveratrol supplementation in irradiated mice on the induction of micronuclei in peripheral blood and bone marrow reticulocytes. Mutagenesis.

[101] Schwartz L.B., Austen K.F., Wasserman S.I. (1979). Immunologic release of beta-hexosaminidase and beta-glucuronidase from pu-rified rat serosal mast cells. J. Immunol..

