# Supplementary material for: Resveratrol Treatment Prevents Increase of Mast Cells in Both Murine OVA Enteritis and IL-10−/− Colitis
Source: Int J Mol Sci. 2022 Jan 21;23(3):1213. doi: 10.3390/ijms23031213 (PMC8836010; doi:10.3390/ijms23031213)
Supplement: Supplementary file 1 [file ijms-23-01213-s001.zip › ijms-1544247-supplementary.pdf]

Figure S1

A

Stool score (OVA)						
p-Value	C + W	C + E	C + R	O + W	O + E	O + R
C + W	-	-	-	-	-	-
C + E	ns	-	-	-	-	-
C + R	ns	ns	-	-	-	-
O + W	< 0.01	< 0.01	< 0.001	-	-	-
O + E	ns	ns	ns	ns	-	-
O + R	ns	ns	< 0.05	ns	ns	-

B

Tissue damage (OVA)						
p-Value	C + W	C + E	C + R	O + W	O + E	O + R
C + W	-	-	-	-	-	-
C + E	ns	-	-	-	-	-
C + R	ns	ns	-	-	-	-
O + W	< 0.001	< 0.01	< 0.01	-	-	-
O + E	< 0.01	ns	ns	ns	-	-
O + R	< 0.05	ns	ns	ns	ns	-

Figure S1. P-values corresponding to Figure 1 showing stool score (**A**) and tissue damage (**B**) in OVA-treated mice. Mice received water (W), 50 mg/kg bodyweight (BW) ethanol (E) or 50 mg/kg BW resveratrol (R) via drinking water for 28 days and were intraperitoneally (i.p.) sensitized with 50 µg Ovalbumin (OVA, (O)) in alum (1:2) on day 5 and 11 and further treated with or without (control, (C)) 50 mg OVA orally on day 15, 18, 20, 22, 24 and 27. Group size was n = 8, respectively.

Figure S2

A

MC/mm² in Duodenum (OVA)						
p-Value	C + W	C + E	C + R	O + W	O + E	O + R
C + W	-	-	-	-	-	-
C + E	ns	-	-	-	-	-
C + R	ns	ns	-	-	-	-
O + W	< 0.01	ns	< 0.01	-	-	-
O + E	< 0.001	< 0.01	< 0.001	ns	-	-
O + R	ns	ns	ns	ns	< 0.001	-

B

MC/mm² in Colon (OVA)						
p-Value	C + W	C + E	C + R	O + W	O + E	O + R
C + W	-	-	-	-	-	-
C + E	ns	-	-	-	-	-
C + R	ns	ns	-	-	-	-
O + W	< 0.01	< 0.01	< 0.001	-	-	-
O + E	< 0.001	< 0.01	< 0.001	ns	-	-
O + R	ns	ns	ns	< 0.05	< 0.001	-

C

Relative *Mcpt4* mRNA expression (OVA)						
p-Value	C + W	C + E	C + R	O + W	O + E	O + R
C + W	-	-	-	-	-	-
C + E	ns	-	-	-	-	-
C + R	ns	ns	-	-	-	-
O + W	ns	ns	< 0.05	-	-	-
O + E	ns	ns	ns	ns	-	-
O + R	ns	ns	ns	ns	ns	-

D

Relative *Mc-cpa* mRNA expression (OVA)						
p-Value	C + W	C + E	C + R	O + W	O + E	O + R
C + W	-	-	-	-	-	-
C + E	ns	-	-	-	-	-
C + R	ns	ns	-	-	-	-
O + W	< 0.05	< 0.05	< 0.05	-	-	-
O + E	ns	ns	ns	ns	-	-
O + R	ns	ns	ns	ns	ns	-

E

Relative *Il3rα* mRNA expression (OVA)						
p-Value	C + W	C + E	C + R	O + W	O + E	O + R
C + W	-	-	-	-	-	-
C + E	ns	-	-	-	-	-
C + R	ns	ns	-	-	-	-
O + W	ns	< 0.05	< 0.05	-	-	-
O + E	ns	ns	ns	ns	-	-
O + R	ns	ns	ns	< 0.05	ns	-

Figure S2. P-values corresponding to Figure 2 showing MC numbers in duodenum (**A**) and colon (**B**), relative *Mcpt4* (**C**), *Mc-cpa* (**D**) and *Il3rα* (**E**) mRNA expression in OVA-treated mice. Mice received water (W), 50 mg/kg bodyweight (BW) ethanol (E) or 50 mg/kg BW resveratrol (R) via drinking water for 28 days and were intraperitoneally (i.p.) sensitized with 50 µg Ovalbumin (OVA, (O)) in alum (1:2) on day 5 and 11 and further treated with or without (control, (C)) 50 mg OVA orally on day 15, 18, 20, 22, 24 and 27. Group size was n = 8, respectively.

Figure S3

A

MC/mm² in Duodenum (IL-10 -/-)						
***p*-Value**	C + W	C + E	C + R	IL-10 -/- + W	IL-10 -/- + E	IL-10 -/- + R
C + W	-	-	-	-	-	-
C + E	ns	-	-	-	-	-
C + R	ns	ns	-	-	-	-
IL-10 -/- + W	< 0.01	< 0.01	< 0.01	-	-	-
IL-10 -/- + E	< 0.05	< 0.05	< 0.01	ns	-	-
IL-10 -/- + R	ns	ns	ns	< 0.01	< 0.05	-

B

MC/mm² in Colon (IL-10 -/-)						
***p*-Value**	C + W	C + E	C + R	IL-10 -/- + W	IL-10 -/- + E	IL-10 -/- + R
C + W	-	-	-	-	-	-
C + E	ns	-	-	-	-	-
C + R	ns	ns	-	-	-	-
IL-10 -/- + W	ns	ns	ns	-	-	-
IL-10 -/- + E	< 0.01	< 0.05	< 0.01	ns	-	-
IL-10 -/- + R	ns	ns	ns	ns	< 0.05	-

C

Tissue damage (IL-10 -/-)						
***p*-Value**	C + W	C + E	C + R	IL-10 -/- + W	IL-10 -/- + E	IL-10 -/- + R
C + W	-	-	-	-	-	-
C + E	ns	-	-	-	-	-
C + R	ns	ns	-	-	-	-
IL-10 -/- + W	< 0.001	< 0.01	< 0.01	-	-	-
IL-10 -/- + E	< 0.001	< 0.001	< 0.01	ns	-	-
IL-10 -/- + R	< 0.05	ns	ns	ns	ns	-

D

Goblet cells (IL-10 -/-)						
***p*-Value**	C + W	C + E	C + R	IL-10 -/- + W	IL-10 -/- + E	IL-10 -/- + R
C + W	-	-	-	-	-	-
C + E	ns	-	-	-	-	-
C + R	ns	ns	-	-	-	-
IL-10 -/- + W	< 0.05	< 0.05	< 0.05	-	-	-
IL-10 -/- + E	< 0.01	< 0.01	< 0.01	ns	-	-
IL-10 -/- + R	ns	ns	ns	ns	ns	-

E

Cell infiltration (IL-10 -/-)						
***p*-Value**	C + W	C + E	C + R	IL-10 -/- + W	IL-10 -/- + E	IL-10 -/- + R
C + W	-	-	-	-	-	-
C + E	ns	-	-	-	-	-
C + R	ns	ns	-	-	-	-
IL-10 -/- + W	< 0.01	< 0.01	< 0.01	-	-	-
IL-10 -/- + E	< 0.01	< 0.01	< 0.001	ns	-	-
IL-10 -/- + R	ns	ns	< 0.05	ns	ns	-

Figure S3. P-values corresponding to Figure 3 showing MC numbers in duodenum (**A**) and colon (**B**), tissue damage (**C**), goblet cells (**D**) and cell infiltration (**E**). Mice received water (W), 50 mg/kg BW ethanol (E) or 50 mg/kg BW resveratrol (R) via drinking water for 90 days. Group size was n = 5 for wt mice (controls, (C)) and n = 9-10 for IL-10 $^{-/-}$, respectively.

Figure S4

A

p-Value	Control + D	IgE/DNP	IgE/DNP + D	IgE/DNP + R
	**β-Hexosaminidase release**			
Control + D	-	-	-	-
IgE/DNP	< 0,0001	-	-	-
IgE/DNP + D	< 0,0001	0,4262	-	-
IgE/DNP + R	0,0529	< 0,0001	< 0,0001	-

B

p-Value	Control + D	IgE/DNP	IgE/DNP + D	IgE/DNP + R
	Ccl2 **relative mRNA expression [% of stimulated control]**			
Control + D	-	-	-	-
IgE/DNP	0,0145	-	-	-
IgE/DNP + D	0,0033	0,2328	-	-
IgE/DNP + R	0,7206	0,0024	0,0271	-

C

p-Value	Control + D	IgE/DNP	IgE/DNP + D	IgE/DNP + R
	Tnf-α **relative mRNA expression [% of stimulated control]**			
Control + D	-	-	-	-
IgE/DNP	0,004	-	-	-
IgE/DNP + D	< 0,0001	0,0166	-	-
IgE/DNP + R	0,707	0,0095	< 0,0001	-

D

p-Value	Control + D	LPS	LPS + D	LPS + R
	Tnf-α **relative mRNA expression [% of stimulated control]**			
Control + D	-	-	-	-
LPS	< 0,0001	-	-	-
LPS + D	< 0,0001	0,1047	-	-
LPS + R	0,5884	< 0,0001	< 0,0001	-

Figure S4. P-values corresponding to Figure 4 showing β-hexosaminidase release and relative mRNA expression of *Ccl2* and *Tnf-α* in IgE/2,4-dinitrophenyl (DNP)- as well as *Tnf-α* in lipopolysaccharide (LPS)-stimulated bone marrow-derived mast cells (BMMC) treated with resveratrol. Cells were incubated with 50 µM resveratrol (R) or the corresponding vehicle control DMSO (D) for 60 min prior to DNP-specific IgE treatment for 60 min and subsequent stimulation with 10 µg/ml DNP for 30 min to determine β-hexosaminidase release (**A**) (n = 14) and 90 min for mRNA expression of *Ccl2* (**B**) (n = 3) and *Tnf-α* (**C**) (n = 10). For LPS stimulation, cells were incubated with 50 µM resveratrol or the corresponding control DMSO for 60 min prior to treatment with 1 µg/ml LPS for 3h to determine *Tnf-α* mRNA expression (**D**) (n = 13).

Figure S5

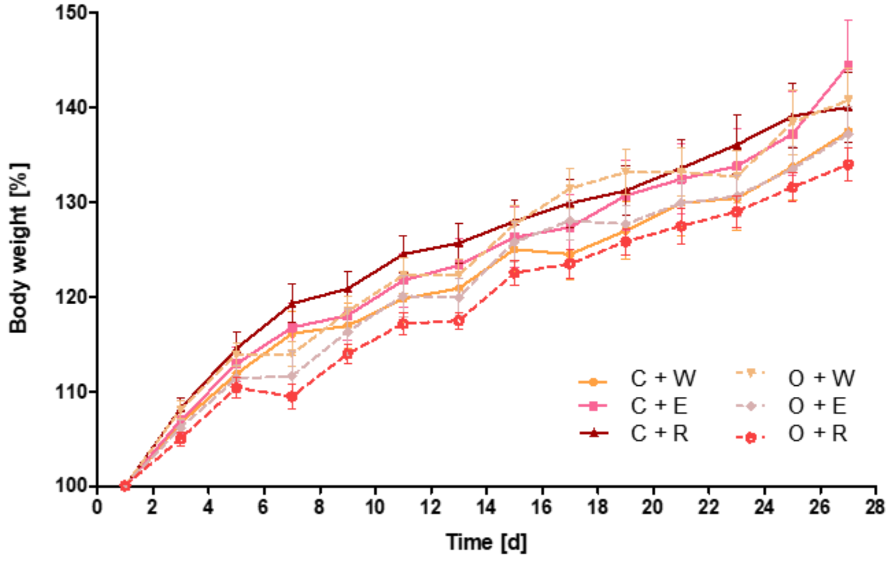

Figure S5. Relative body weight course in control mice and Ovalbumin (OVA, O) treated mice for 28 days. Mice received water (W), 50 mg/kg bodyweight (BW) ethanol (E) or 50 mg/kg BW resveratrol(R) via drinking water for 28 days and were intraperitoneally (i.p.) sensitized with 50 µg OVA in alum (1:2) on day 5 and 11 and further treated with or without (control, (C)) 50 mg OVA orally on day 15, 18, 20, 22, 24 and 27. Weight was monitored every second day.

Figure S6

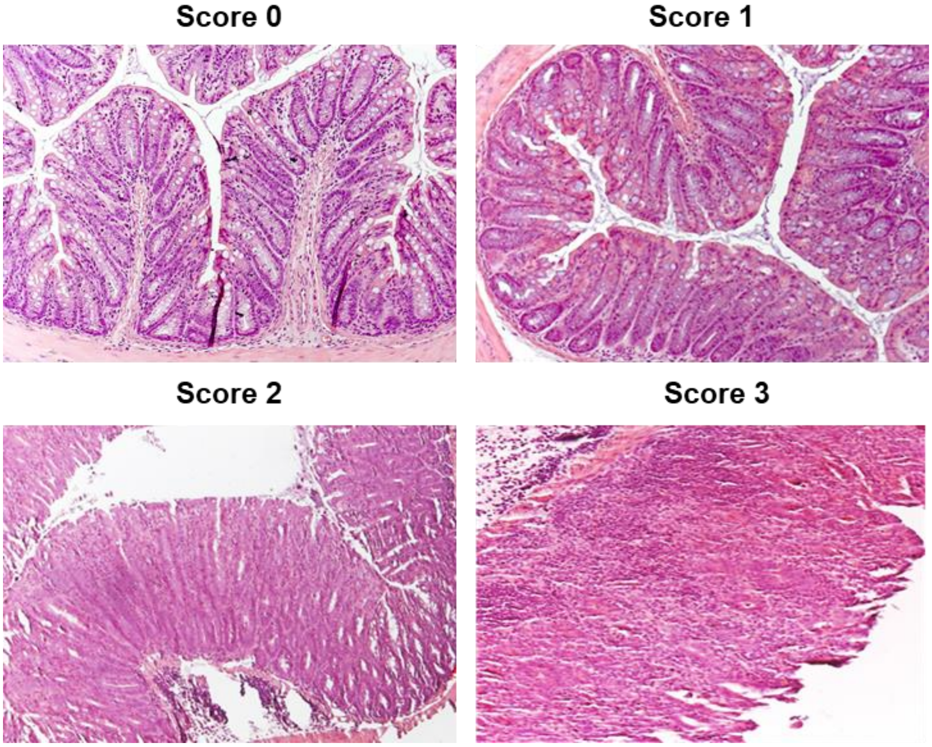

Figure S6. Representative pictures of colon tissue from IL-10 $^{-/-}$ mice treated with resveratrol for analyzing severity of colitis captured as tissue damage, goblet cells and cell infiltration with scores ranging from 0 to 3. Scores were determined in at least three hematoxylin- and eosin-stained tissue sections. Scores contained the following criteria for tissue damage (score 0: undamaged mucosa, 1: single lymphoepithelial damages, 2: surface damages of mucosa, 3: extensive mucosal damage and damage of deeper structures of the intestinal wall), number of goblet cells (score 0: normal; score 1: <50% reduction; score 2: 50-90% reduction; score 3: >90% reduction), and infiltration of inflammatory cells (score 0: low numbers of inflammatory cells in lamina propria; score 1: increased number of inflammatory cells in the lamina propria; score 2: accumulation of inflammatory cells in the lamina propria and infiltration into the submucosa; 3: transmural distribution of inflammatory cells).
